# Space telescope set to open a new chapter in China's solar research

**DOI:** 10.1093/nsr/nwad289

**Published:** 2023-12-11

**Authors:** Ling Xin

**Affiliations:** Ling Xin is a science writer based in Ohio, USA

One year after China launched its first dedicated space telescope to study the Sun, the Advanced Space-based Solar Observatory (ASO-S) is now fine-tuned and ready for discoveries during the activity peak of this solar cycle. ‘ASO-S has completed its in-orbit tests, and all instruments are in stable operation. Some early results are already in the pipeline,’ said the mission's chief scientist, Gan Weiqun, as the telescope was officially delivered to his co-workers at the Purple Mountain Observatories, the Chinese Academy of Sciences (CAS) in Nanjing, the main user institution of ASO-S, on 25 September, 2023.

When the spacecraft carrying ASO-S lifted off from the Jiuquan Satellite Launch Center in northwestern China's Gobi Desert on 9 October 2022, it was a dream come true for generations of solar researchers in China. For the first time, they would have their own facility to systematically observe our nearest star during the upcoming solar maximum, and hopefully be the start of a line of world-leading probes and observation data in years to come.

## LATECOMER TO SOLAR CLUB

Solar physics has been a major branch of astronomy research in China, Gan said. Back in 2010, the country was already one of the world's largest publishers of papers in solar physics—second only to the USA. However, nearly all space observation data used in those papers came from telescopes developed by the USA, Europe or Japan.

For a long time, Chinese researchers’ attempts to build their own solar probe did not work out. Different ideas have been proposed since the mid-1970s, but only one of them made its way into orbit: a gamma-ray monitor launched on board the Shenzhou-2 spacecraft in 2001. The monitor, which contained three high-energy radiation spectrometers and took 7 years to develop, detected X-ray and gamma-ray bursts of multiple solar flares during its 6-month run in space.

When the concept of ASO-S first emerged in 2011, nearly 70 telescopes had been put into orbit to study the Sun around the world. ‘Apparently we were lagging behind, and a key task facing the Chinese solar community was to come up with something that was both technically feasible and scientifically unique,’ Gan said.

By the time the mission was given the green light 6 years later, they had decided on what they wanted to do with the telescope: to simultaneously monitor the two most violent activities on the Sun—solar flares and coronal mass ejections—as well as the magnetic field that is believed to be driving these eruptions. By better understanding the links between these phenomena, researchers hoped to find out exactly how they trigger hazardous conditions in space that can knock out satellite services and power grids on Earth.

To achieve this goal, the 857-kg ASO-S would be equipped with three instruments: the Hard X-ray Imager (HXI), the Lyman-alpha Solar Telescope (LST) and the Full-disk vector MagnetoGraph (FMG), which are used to observe the solar flares, coronal mass ejections and the solar magnetic field, respectively.

## ONE YEAR IN ORBIT

After a scientific satellite reaches orbit, it needs to go through a series of testing and calibration to improve the quality of its images, which is often affected by environmental factors and the telescope itself. ‘Calibration can take a long time, and the outcome of calibration largely determines the scientific value of your data,’ Gan said.

**Figure 1.**
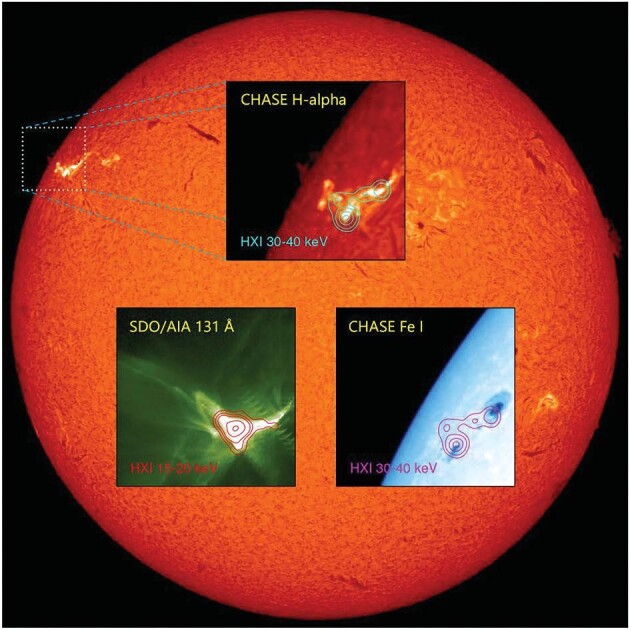
Hard X-ray image observed by HXI (contours) on board ASO-S overlaid on images observed by CHASE and SDO. *(Courtesy of Prof. Weiqun Gan)*

As Gan's team focused on fine-tuning ASO-S in the past year, the three instruments snapped images to show how well they worked compared with designed performances. The biggest surprise came from HXI, Gan said, which aimed to study how electrons are accelerated in solar flares through indirect imaging in the energy range between 30 and 200 keV (Fig. 1).

‘We had never built such an instrument before, and there was no good way to test it on the ground,’ he said. When HXI was first switched on from orbit on 18 October 2022, no strong solar flares were detected. It was not until 3 weeks later that the first flare was observed, and the image reconstructed later using the X-ray measurements and algorithms was exactly what they were expecting. ‘That was a huge relief. It means both our hardware and software worked,’ he said.

By October this year, HXI had spotted >300 solar flares and the quality of its images far exceeded expectations, making detections over a wider energy range between 15 and 300 keV, and revealing more structural details than NASA’s Reuven Ramaty High Energy Solar Spectroscopic Imager mission had to show about the flares, according to Gan.

Research using the HXI images is already under way, including collaborative work with international partners from the University of Applied Sciences Northwestern Switzerland, who are also using the hard X-ray imager onboard European Space Agency's Solar Orbiter probe.

**Figure 2.**
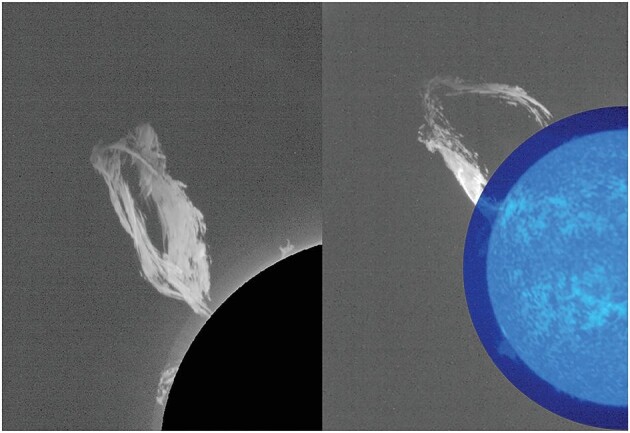
Solar prominences observed by LST on board ASO-S. *(Courtesy of Prof. Weiqun Gan)*

For LST, which is designed to take images of the Sun and its inner corona in both the Lyman-alpha and visible wavebands with high temporo-spatial resolution, its performance has yet to fully meet expectations. Researchers were working hard to pin down the problem and look for ways to make up for the gap, Gan said (Fig. 2).

Marco Romoli, from the University of Firenze in Italy, said that LST has two capabilities that are novel compared with the coronagraph METIS on Solar Orbiter and all previous coronagraphs. Built as an internally occulted coronagraph, LST is the first of this kind to observe the ultraviolet light produced by the hydrogen in the solar corona and it allows the observation of the solar corona at <1.7 solar radii, Romoli said.

Despite existing problems, LST had recorded a considerable number of events with observations that had never been done before, Gan said. Romoli's team was helping shape the first scientific results from LST. ‘Our partnership started on the instrument level many years ago. I’m on the scientific committee of ASO-S, and the LST team members are contributing to METIS science,’ Romoli said.

Work on FMG was also focused on narrowing the gap between the actual and designed performances of the instrument, which measures the magnetic fields of the entire solar disk with high spatial and temporal resolutions. ‘At least, we can achieve the designed parameters in principle by sacrificing a small amount of temporal resolution,’ Gan said.

A collection of the early research results of the ASO-S mission would be published in the *International Journal of Solar Physics* early next year, he said.

## SOLAR CYCLE 25

Although our observation of the universe now extends to 13 billion light years away, the Sun is still the only star that we can study in detail, and fundamental questions remain unanswered, Gan said. For instance, the Sun's explosions are thought to be powered by its magnetic fields. However, what kind of magnetic field triggers flares, or coronal mass ejections, or both, and how do its shape and structure determine the level of an explosion? Unraveling these mysteries may make the prediction of space weather possible—just by monitoring the Sun's magnetic fields.

In Solar Cycle 25, for the first time, the Sun is being watched by a number of space- and ground-based observatories from various points of view, Romoli said. On the ground, the new 4-m-diameter Daniel K. Inouye Solar Telescope in Hawaii is starting to become fully operational. From space, in the Earth's neighborhood, there are NASA’s multi-wavelength Solar Dynamical Observatory (SDO) and the ESA–NASA Solar and Heliospheric Observatory, launched in 1995 and still partially operational. Of course, the new flagship missions in solar orbit are watching the Sun from close by: NASA’s Parker Solar Probe will get the closest ever to the Sun at ∼10 solar radii to measure solar wind and energetic particles, while ESA’s Solar Orbiter is the most comprehensive observatory that is orbiting the Sun.

While solar telescopes may have overlapping designs, they are never the same and joint observation is important, Gan said. For instance, SDO can image the Sun in 10 different wavebands, but it does not work in the Lyman-alpha band or the X-rays, and is highly complementary with ASO-S. So is the newly launched Indian solar satellite Aditya-L1 to ASO-S.

The designed life span for ASO-S is 4 years. It is expected to cover at least the current solar cycle, and maybe even the next cycle—the longer it runs, the better, Gan said. ‘Technically, if a satellite's life span is above four years, it calls for much higher manufacturing standards and the building costs increase significantly. So we had compromises to make,’ he said.

As for China's next space-based solar observatory, Gan envisioned it to be world-leading. Right now, two candidates being considered are the Solar Polar-orbit Observatory and the Lagrange 5 Point Exploration mission. They are designed to operate from places that no other solar telescopes have been to, he said. The solar community is also open to proposals with technical innovations, such as formation flying of two spacecrafts to obtain images of the Sun with unprecedented spatial resolution. ‘There's still a long way to go and a lot to do before we decide what we’ll go for after the ASO-S mission,’ Gan said.

